# Design of a graphical and interactive interface for facilitating access to drug contraindications, cautions for use, interactions and adverse effects

**DOI:** 10.1186/1472-6947-8-21

**Published:** 2008-06-02

**Authors:** Jean-Baptiste Lamy, Alain Venot, Avner Bar-Hen, Patrick Ouvrard, Catherine Duclos

**Affiliations:** 1 Laboratoire d'Informatique Médicale et de Bioinformatique (LIM&BIO), UFR SMBH, Université Paris 13, 74 rue Marcel Cachin, 93017 Bobigny cedex, France; 2 Société de Formation Thérapeutique du Généraliste (SFTG), 233 bis rue de Tolbiac, 75013 Paris, France

## Abstract

**Background:**

Drug iatrogeny is important but could be decreased if contraindications, cautions for use, drug interactions and adverse effects of drugs described in drug monographs were taken into account. However, the physician's time is limited during consultations, and this information is often not consulted. We describe here the design of "Mister VCM", a graphical interface based on the VCM graphical language, facilitating access to drug monographs. We also provide an assessment of the usability of this interface.

**Methods:**

The "Mister VCM" interface was designed by dividing the screen into two parts: a graphical interactive one including VCM icons and synthetizing drug properties, a textual one presenting on demand drug monograph excerpts. The interface was evaluated over 11 volunteer general practitioners, trained in the use of "Mister VCM". They were asked to answer clinical questions related to fictitious randomly generated drug monographs, using a textual interface or "Mister VCM". When answering the questions, correctness of the responses and response time were recorded.

**Results:**

"Mister VCM" is an interactive interface that displays VCM icons organized around an anatomical diagram of the human body with additional mental, etiological and physiological areas. Textual excerpts of the drug monograph can be displayed by clicking on the VCM icons. The interface can explicitly represent information implicit in the drug monograph, such as the absence of a given contraindication. Physicians made fewer errors with "Mister VCM" than with text (factor of 1.7; *p *= 0.034) and responded to questions 2.2 times faster (*p *< 0.001). The time gain with "Mister VCM" was greater for long monographs and questions with implicit replies.

**Conclusion:**

"Mister VCM" seems to be a promising interface for accessing drug monographs. Similar interfaces could be developed for other medical domains, such as electronic patient records.

## Background

When prescribing a drug, the physician needs to ensure that the prescription is safe. However, medication errors are frequent and constitute a public health problem [1]. Serious events reported to the FDA (Food and Drug Administration) increased 4 times faster than the total number of outpatient prescriptions. In the prescription process, the physician must first decide whether he is sufficiently familiar with the various contraindications, drug interactions and cautions for use. If not, he must consult the drug monograph. It may take too long to read this monograph in full if the text is long, and this reading requires a cognitive effort. For example, if the physician wants to know whether the drug is contraindicated in case of renal failure, he must read the contraindication section of the drug monograph until he finds the necessary information. If the drug is not contraindicated, this knowledge is implicit, and the whole section must be read before the physician can deduce that there is no contraindication.

Graphical user interfaces (GUIs) have been widely studied in computer science [2,3], and have been successfully used to improve the presentation of medical data or knowledge or to facilitate access to this information [4-6]. Various kinds of graphical interfaces have been proposed for drug monographs.

Infobuttons [7] have been proposed as a way of integrating context-sensitive knowledge into the prescription workflow. They can be used to display an extract of the drug monograph at the appropriate moment. However, infobuttons provide no information unless the physicians click on them and the physicians must still read a certain amount of text without being sure this text is relevant.

Hypertextual tables of contents are a common tool for navigating through the various sections of the drug monograph (*e.g. *contraindications or adverse effects). However, the physician must still read the whole section at the end of the navigation. Some tables of contents may be developed in more detail. For example, the contraindications section may contain "cardiac contraindications" and "pulmonary contraindications" subsections. However, detailed tables of contents require a large number of mouse clicks, making the navigation tedious, especially when the knowledge sought is implicit.

In this context, it would be worthwhile to design new tools helping physicians (i) to determine whether they need to consult the drug monograph and, (ii) to select the part of the text corresponding to the patient concerned, in order to simplify the cognitive tasks required for safe prescription. Our approach included two steps. We first built a graphical language called VCM (*Visualisation des Connaissances Médicales*; visualization of medical knowledge) [8]. In a second time, we have used VCM icons to develop a graphical interactive interface, called "Mister VCM", which is presented here. This interface is based on an anatomical diagram of the human body on which icons may be placed. It conveys medical information even before any interaction, such interactions with the interface being required only to obtain more detailed knowledge about the drugs than is initially presented. This paper also describes a preliminary evaluation of the usability of this interface.

## Methods

### Design and implementation of "Mister VCM"

The approach consisted in (i) distinguishing a first, graphical, part of the screen that displays synthetical information about drug properties, on which the physician can interact in order to display drug monograph excerpts in a second, textual, part, (ii) dividing the graphical part in three zones, respectively for contraindications, cautions for use, and adverse effects, (iii) drawing an anatomical schema in each of the three zones, and (iv) representing the drug properties by adding interactive icons on these anatomical schemas; when one of these icons is clicked, the corresponding excerpts in the drug monograph is displayed in the textual part of the interface.

The following steps has been pursued for designing the interface of "Mister VCM":

#### Using a graphical overview + detail design

An overview + detail design model was used, which consists in dividing the interface into two parts: the first one is a global overview and the second one displays on-demand the complete details of the elements the user has selected in the overview. In "Mister VCM", the detail part displays textual drug monograph excerpts.

#### Grouping the drug information according to its medical use

The information in the sections of the drug monograph we selected can be categorized in three groups : (1) the information relevant to determine whether the drug can be prescribed to the patient (absolute contraindications with both diseases and drugs), (2) the information related to the specific cautions or follow-up procedures that have to be considered for the patient, such as a renal surveillance for an aged patient (cautions for use, relative contraindications with diseases or drugs), and (3) the information relevant for the patient follow-up (adverse effects).

The graphical overview is divided into three similar parts, one for each group.

#### Identifying and classifying the concepts contained in drug information

The various concepts that can be involved in contraindications, drug interactions, cautions for use and adverse effects, including diseases, drugs, procedures, patient characteristics *e.g. *age, or lifestyle, *e.g. *alcohol consumption, were determined using our expertise and published models [9].

These concepts are very numerous and have been classified to facilitate their spatial grouping according to specific axes, *e.g. *anatomy, etiology. The following rules were taken into account for determining these axes: (a) they should be commonly used in medical classifications for classifying diseases or drugs, (b) they should be commonly used and well-understood by physicians, and (c) any disease, drug, test or patient characteristic present in drug monographs should be classified in at least one axis.

Each of the three parts of the graphical overview was divided into zones used for representing separately each axis.

#### Translating the medical concepts graphically

The concepts have been graphically represented using the icons of the VCM language [8], that we initially developed for the representation of the various contraindications, drug interactions, cautions for use and adverse effects of drugs.

#### Using an anatomical schema divided into several areas for representing the anatomic axis

The number of anatomic locations contained in the various concepts is very high. Consequently, we decided to divide the anatomic zone into several areas organized spatially according to an anatomical schema, forming the outline of a human body. The anatomical schema must satisfy the following requirements: (a) be able to represent all the anatomical structures on a single two-dimensional schema, (b) give to each anatomical location a space of the same size, which prevents the physician from interpreting the size as, for example, the importance of a contraindication, and (c) be able to receive VCM icons on the various anatomical locations. These requirements are such that the schema cannot be realistic, since true organs are organized in 3D and they have diverse sizes.

Two criteria were considered for choosing the exact position of a given anatomic structure on the anatomical representation: (a) the real location of the anatomical structure, *e.g. *mouth is located at the bottom of the head, and (b) the location of the other anatomical structures, such that they can be organized coherently, *e.g. *the various organs that compose the digestive tract should be organized together.

#### Implementation method

A prototype of "Mister VCM" has been implemented using the *PYTHON *programming language , and *XML*, *HTML *and *JAVASCRIPT *technologies. The prototype acts like a web server, and generates web pages including VCM icons and "Mister VCM" that can be viewed in a web browser. Drug monographs must be encoded in *XML *before they can be visualized by the software. The encoding was performed automatically using the indexed knowledge contained in the French drug database named Theriaque [10], after a manual mapping of the Theriaque thesaurus to VCM.

In the Theriaque database, drug monographs are split into term-indexed paragraphs. A paragraph is represented on the first "Mister VCM" if it expresses absolute contraindications, on the second one if it expresses relative contraindications or caution for use, and on the third one if it expresses adverse effects. Then, each indexed term in the paragraph is translated into a VCM icon; if no VCM icon is available for a given term, the icon associated to its parent is used. For each anatomical location, functional location and etiology present on this VCM icon, the associated location on "Mister VCM" is considered. If this location is empty, the VCM icon is placed there. If there is already another icon there, it is replaced by the smaller common parent icon of the previous icon and the new one, and a shadow is added to the icon. This shadow indicates that the icon is the result of the combination of several icons (see example on figure 2 for allergy). Finally, any location on "Mister VCM" on which no icon was placed is grayed.

The rules for merging icons are based on *is-a-kind-of *relations defined between the VCM icons: the rule consists in using the more precise common parent of the icons involved, *e.g. *the icons for heart failure and heart rhythm disorders can be merged into the icon for cardiac disease. The locations on "Mister VCM" cover the first digit of ICD10 and ATC classifications ensuring the existence of this general concept, so that every disease or drug icons can be placed somewhere on "Mister VCM". In addition, VCM represents a drug by an icon combining the icon of the disease it treats and a green upright pictogram indicating "treatment". Consequently, drug icons can be positioned on the anatomical axis and mixed with disease icons, and it is possible to represent contraindications and drug interactions on the same "Mister VCM" (in figure 2, "Mister VCM" displays both VCM icons for diseases, for example viral infection, and VCM icons for drugs, like antitumorals).

### Evaluation

The objective was to evaluate the usability of "Mister VCM" for physicians: Does a physician understand the interface? Does a physician manage to interact with the interface for obtaining drug information? We compared the correctness of the responses, the response time and the satisfaction obtained when general practitioners sought an answer to a medical question in a drug monograph, using either a textual interface or a graphical interface with "Mister VCM".

#### Evaluator recruitment

The evaluators were French general practitioners recruited from the SFTG (*Société de Formation Thérapeutique du Généraliste*), an association responsible for the ongoing training of doctors throughout their careers. The evaluation took place on the same day as the evaluation of the VCM language [8], and involved the same 11 general practitioners. They had been trained in the use of VCM and "Mister VCM" during an initial meeting of 2 hours, and a personal work on a training software ranging from 2 to 7 hours.

#### Design of evaluation interfaces

For the purpose of evaluation, two interfaces were designed. The first, the textual interface, displayed the text of the drug monograph, using a scrollbar when the text did not fit on the screen. The text was divided into four sections: contraindications, drug interactions, cautions for use and adverse effects. This is the usual presentation for drug monographs in paper, and also in many electronic, desktop references.

The second interface, the graphical interface, was composed of three "Mister VCM". contraindications and drug interactions were merged in the first "Mister VCM", and cautions for use were represented on the second and adverse effects third. The "Mister VCM" were interactive and were able to display textual excerpts of the drug monographs. For a given drug monograph, the textual content of both interfaces was the same, *i.e. *the sum of all textual excerpts available in the graphical interface were identical to the text displayed in the textual interface.

#### Documents and questions

Fictitious drug monographs were used, so that physicians could not rely on their memory. They were created by randomly mixing the paragraphs of 15 randomly-chosen real drug monographs. Only contraindication, drug interaction, caution for use and adverse effect sections were considered. The paragraphs were exactly as contained in the drug monograph, without any modification or simplification. The drug monographs we used came from the French database Theriaque [10].

Questions were randomly-generated yes/no questions of the form "Can this drug be prescribed without precaution to a patient suffering from disease X/taking drug Y?" or "Can this drug cause adverse effect Z?". Two question types were considered: questions with an explicit reply, and questions with implicit reply, for which the physician has to read the whole text before deducing the response. There were 10 short monographs consisting of 10 paragraphs and 10 long monographs consisting of 30 paragraphs, each of these two groups including 5 questions of each type.

Documents and questions were randomly generated with as few interventions on the part of the evaluation designers as possible. A paragraph database was first created by extracting all the paragraphs expressing contraindications, cautions for use, drug interactions or adverse effects from a random set of drug monographs, selected from the entire Theriaque database.

Documents were created by concatenating random paragraphs from the base (without duplicates), in random order. In each document, paragraphs expressing contradictory information were manually replaced by new random ones. Questions were related to the content of a paragraph, randomly chosen in the document (for questions with an explicit response) or from the other paragraphs in the database (for questions with an implicit response).

The *PYTHON *programming language was used to design the software drawing "Mister VCM" from Theriaque indexed data.

#### Design of the interface usability evaluation

We asked physicians to find the answers to medical questions by deduction from the reading of documents (described above) and measured their response times. Each question was associated with a different document, and was asked twice: once with the document displayed on a graphical interface using "Mister VCM", and once with the document displayed on a textual interface, in a per-evaluator random order.

The evaluation was divided into two sequences separated by a pause of 15 minutes. Each sequence included each question once, with either the graphical or textual interface. During the evaluation, the question was displayed and the evaluator was asked to click on a button to display the document, and then to click on the response. The response time was recorded by the computer, as well as the accuracy of the response (either right or wrong). Physicians were told that all questions and documents were different, although this was not actually the case. They were also told not to waste time between the document appearing on screen and giving their answer.

The severity of the errors done by the physicians were difficult to determine, especially since fictitious monographs were used. For question relating to contraindications or drug interactions, we took into account the level of contraindication to classify errors in three categories: error leading to (1) prescribe a drug that is absolutely contraindicated, (2) prescribe a drug without taking into account a caution for use, and (3) to not prescribe a drug that could have been prescribed.

At the end of the evaluation, evaluators were asked to indicate the interface with which they feel they have replied (a) more rapidly and (b) more accurately, in a one-line free-text field; these questions measured the evaluator's subjective perception of time and of the outcomes, and they allowed evaluation of whether the physicians were more confident when using "Mister VCM". The accuracy, the response time and the questions about physicians' opinions correspond to the three components of usability: respectively effectiveness, efficiency and satisfaction, as proposed by Hornbæk *et al*. [11].

This evaluation was performed on Macintosh computers of equivalent performance, running MacOSX.

#### Statistical methods

For comparing the response times obtained with text and "Mister VCM", we considered three factors: evaluator, document length and question type (*i.e. *with explicit or implicit response). ANOVA was used to investigate the effect of these three factors and their interactions on response time. Paired t-tests were carried out for comparing differences in mean response time.

For comparing the number of errors with text and "Mister VCM", Fisher's exact test was used. For comparing the repartition of the errors in the three categories, for textual and graphical interfaces, Pearson's Chi-squared test was used.

Linear regression analysis was carried out to investigate the relationships between response time and percentage errors, and to take the three factors into account.

The significance threshold was set at α = 0.05. Data were analyzed with R software version 2.2.1 [12].

## Results

### Description of "Mister VCM"

The interface includes three "Mister VCM", one for absolute contraindications, one for the cautions for use and one for adverse effects. "Mister VCM" includes an anatomical diagram for representing the anatomical axis. The etiological axis is represented outside the anatomical schema, as are patient characteristics and life habits.

#### "Mister VCM"'s figure

**Figure 1:**
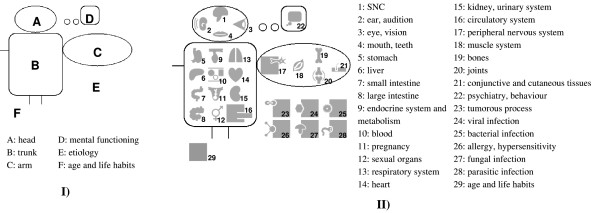
The various areas (I) and anatomical and functional locations and etiologies (II) on "Mister VCM".

"Mister VCM" consists of 6 areas (see figure 1, I). The three first areas compose a schematized human body: the head, the trunk and one arm. The fourth is a bubble on the upper right corner, which represents mental functioning. A fifth area in the lower right corner is devoted to the representation of etiologies. The sixth area, under the schematized body, shows physiological conditions *e.g. *age, and life habits.

#### "Mister VCM" locations

Twenty-one anatomical and functional locations, represented by gray pictograms, are spread over the "Mister VCM" image (see figure 1, II). The anatomical locations on the schema have been chosen using the first digit of the ICD10 and ATC medical classifications, the two being very similar and highly based on anatomy. As a consequence, it covers all the anatomical and functional systems commonly associated with diseases or drugs. Concepts related to a given anatomical structure or to the associated physiological function were placed at the same location, *e.g. *lung infection and respiratory failure. To reduce the number of anatomical and functional locations, some of them have been grouped together, *e.g. *red cells and white cells have been grouped together under the more generic "blood" location.

The various anatomical pictograms have been placed on "Mister VCM" figure according to their approximate real anatomic position, or, when not possible, in a relevant location, *e.g. *the arm contains the musculo-skeletal system. Each anatomic structure is present only once, *e.g. *only one kidney and one bone are represented.

A small gray bubble has been placed in the mental functioning area. In addition, six etiologies, also represented by gray pictograms, have been placed in the etiology area of "Mister VCM".

#### Filling a"Mister VCM"with VCM icons

**Figure 2:**
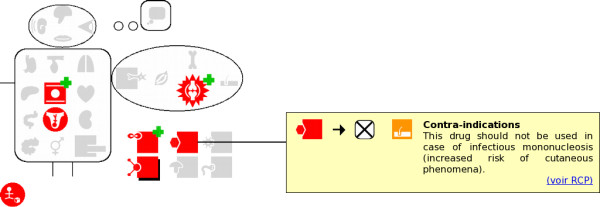
Screenshot of "Mister VCM" displaying contraindications and drug interactions of a penicillin (bacampicillin, 200 mg tablet). Here, the physician has clicked on the VCM icon for viral infection, and the corresponding piece of text is displayed, both in VCM and text.

When a contraindication, drug interaction, caution for use or adverse effect is found in the drug monograph, the corresponding VCM icon is placed over the gray pictogram associated with its anatomical or functional location or etiology. For example, on figure 2 the viral infection pictogram has been replaced by the VCM icon for viral diseases.

When nothing is said about a given anatomical or functional location, the corresponding pictogram is grayed. It must be emphasised that this gray pictogram on "Mister VCM" makes explicit the absence of information related to the anatomical or functional location, *e.g. *a grayed kidney on the contraindication "Mister VCM" indicates that a drug has no renal contraindication. Thus, "Mister VCM" provides a fixed-size rough summary of the drug monograph.

#### Interacting with"Mister VCM"

"Mister VCM" is also an interactive interface, using the *details-on-demand *model [13]. When the physician wants more details about contraindications, drug interactions, cautions for use or adverse effects related to a given anatomical or functional location or etiology, he may click on the corresponding VCM icon to obtain the complete information (the yellow rectangle on the right, in figure 2). The information includes both the corresponding textual excerpt from the drug monograph and a sentence in VCM. When the icon clicked is a composite made by the merging of several icons, the text may be the concatenation of several excerpts from the drug monograph.

### Evaluation results

#### Documents and questions

Short fictitious monographs had a mean of 427 words (for texts) and 15 icons (for "Mister VCM"), and long monographs a mean of 1067 words and 36 icons; this corresponds to a mean of about 29 words per icon.

**Figure 3:**
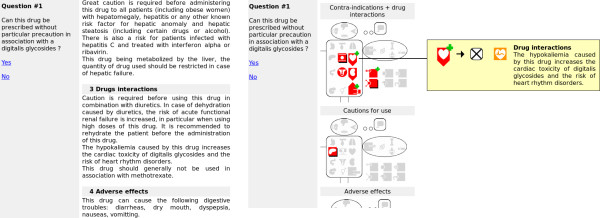
**Screenshots of the evaluation interfaces (extracts).** The two screenshots show the same drug monographs, displayed on the left with the textual interface, and on the right with the graphical interface, after clicking the icon for drugs used for treating heart failure in the "Mister VCM" for contraindications and drug interactions.

Figure 3 shows two screenshots of the evaluation software, using the textual interface and the graphical interface.

#### Correctness of the responses

Correctness of the responses was significantly higher with the graphical interface featuring "Mister VCM" than with the textual interface: 16 errors were recorded with the graphical interface and 27 with the textual one, giving an error ratio of 1.7 in favor of the graphical interface; the difference was significant (*p *= 0.034). The error ratio is 1.0 for short document, and 4.7 for long document. However, one short-document question was asking about adverse effect in case of overdose, and these adverse effects were not presented on the adverse effect "Mister VCM" but in the cautions for use "Mister VCM". This possibly misled the physicians, and many errors were recorded with the graphical interface for this question. Consequently, the 1.0 error ratio for short document should be considered with caution.

With the textual interface, 18 errors related to contraindications or drug interactions. 3 involved absolute contraindications, 6 cautions for use, and 9 led to not prescribe a drug that actually could have been prescribed, the physician thinking wrongly that there was a contraindication. With the graphical interface, 5 errors related to contraindications or drug interactions. 2 involved absolute contraindications, 1 caution for use, and 2 led to not prescribe a non-contraindicated drug. The use of the textual or graphical interfaces has no significant impact on the repartition of the errors in the three category of severity (*p *= 0.52).

Linear regression analysis showed that there was no significant relationship between response time and the correctness of the responses, for either "Mister VCM" or text.

#### Time taken answering questions

**Figure 4:**
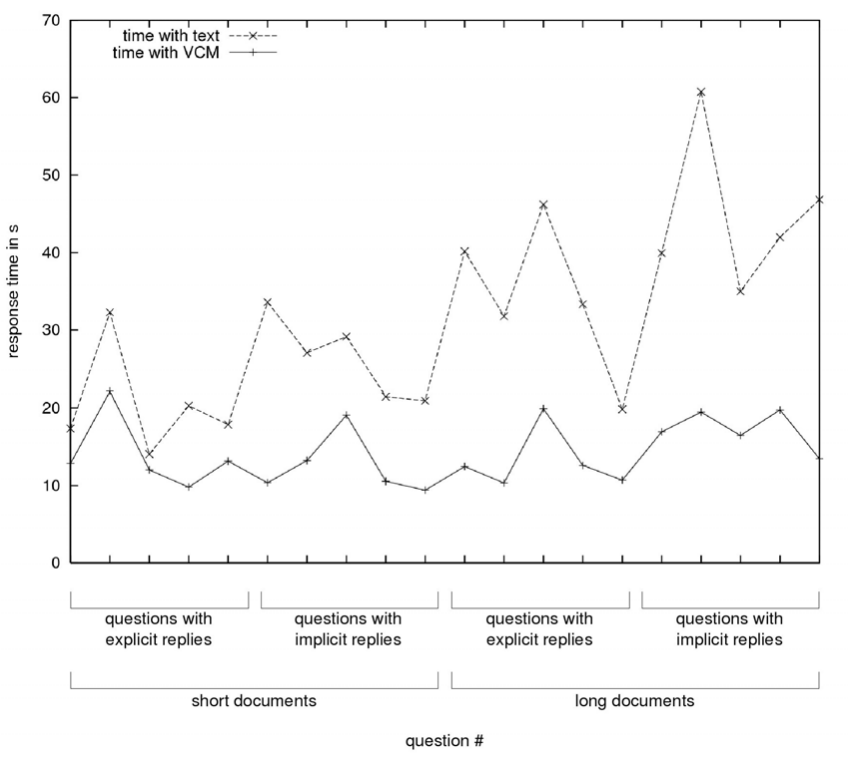
Average response time for questions answered using a graphical interface featuring "Mister VCM" or a textual interface.

All physicians answered more rapidly with "Mister VCM" than with text, with individual text/"Mister VCM" time ratios ranging from 1.3 to 3.9. Figure 4 shows the response times, for the graphical and the textual interfaces. Physicians responded significantly (*p *< 0.001) more rapidly with "Mister VCM" than when consulting the text directly (on average, 2.2 times faster with "Mister VCM").

ANOVA indicated that the question type had an influence on the response time with text (*p *= 0.002), but no influence on the response time with "Mister VCM" (*p *= 0.18). For questions with explicit replies, physicians responded 2.0 times faster with "Mister VCM" than with text, and for questions with implicit replies, 2.4 times faster.

The length of the monograph had a large influence on the response time with text (*p *< 0.001) but a smaller effect on the response time with "Mister VCM" (*p *= 0.04). As the monograph length increases, the response time increases less with "Mister VCM" than with text: between short and long monographs, the average increase in response time observed with "Mister VCM" was only 15%, but 70% with text. "Mister VCM" allowed a time-saving of 70% with short monographs, and 160% with long monographs.

#### Physician satisfaction

After the evaluation, six physicians said they felt that they had answered more accurately with "Mister VCM"; four did not, and one did not reply. Ten physicians said they felt that they had answered more rapidly with "Mister VCM", and one did not reply to this question.

## Discussion

### About "Mister VCM"

"Mister VCM" combines a graphical overview, showing explicitly the presence or the absence of information related to a given anatomical or functional system or etiology, with an interactive interface, allowing rapid access to the complete textual information. The overview of "Mister VCM" shows a graphical representation of a virtual patient with all the clinical problems described in the drug monograph. This overview is not only anatomical and functional, but also summarizes etiological and physiological elements. Drug interactions are included in the absolute contraindications "Mister VCM" in order to facilitate their reading, however it is not destined to replace the drug-drug interaction alert raising systems that are commonly proposed in medical software.

The anatomical part of "Mister VCM" helps organize information concerning numerous diseases and drugs, as the anatomical levels of medical classifications were considered during its design. The anatomical diagram in "Mister VCM" is very stylized: only one arm is detailed and the other limbs are not represented, and organs present in several copies are represented only once. However, with regard to drug knowledge, it is of no value to distinguish *e.g. *the bones in one arm from those in the other arm or the leg, or the left from the right kidney. This may need to be reconsidered for representing patient data in electronic records, or to refine the representation of particular diseases or symptoms, such as atherosclerosis of arteries of the lower extremities.

Several authors have used anatomical diagrams for presenting or entering data in electronic patient records, on an anatomical basis. J. Kirby *et al*. [14] proposed Pen&Pad, commercialized under the name "Clinergy". L. Stoicu-Tivadar *et al*. [15] included an anatomical diagram in their electronic record and reported that general practitioners were interested by it. P.J. McCullagh *et al. *[16] designed an application for multiple sclerosis. Sundvall *et al. *[17] have proposed to use Google Earth for positioning medical documents over a human body. The anatomical schemas found in the literature were designed for entering data in patient records, and therefore they tend to be very realistic, contrasting with "Mister VCM", which was designed for presenting medical knowledge and consequently involves a higher level of abstraction. Some previously published anatomical schemas use several views, *e.g. *for skeleton, muscles, vascular system, and other organs. However, we did not do this for "Mister VCM" so as to avoid the need for multiple images.

As anatomy is insufficient for identifying all diseases and drugs, we added an etiology axis, as in medical classifications which individualize, for example, infectious diseases. In addition, an area is devoted to the representation of physiological data and life habits. No other authors, as far as we are aware, have developed a graphical interface featuring a similar combined use of anatomy, etiology, physiological data and life habits.

"Mister VCM" uses the VCM graphical language, which the physician has to learn; however we have shown that this is possible in six hours [8]. None of the existing anatomical diagrams described in the literature rely on a graphical language. The disease-based approach of VCM is particularly valuable, as it allows drugs to be presented according to the disease they treat; this in turn makes possible to organize drugs on the anatomical schema, and to represent contraindications and drug interactions on the same "Mister VCM". As the consequence, the three "Misters VCM" present on the graphical interface correspond to three levels of importance: absolute contraindications, relative contraindications and cautions for use, and adverse effects.

The detail in the graphical overview of "Mister VCM" can vary according to the complexity of the drug monograph. On "Mister VCM", the graphical representation of a given contraindication is context-dependent since it depends of the presence or the absence of other contraindications related to the same anatomical or functional system. For instance the representation of a contraindication with angina pectoris would be the VCM icon for angina pectoris if there is no other cardiac contraindication, and the more general VCM icon for cardiac diseases if there are other cardiac contraindications. The importance of context-dependent representation of medical information has been stressed by S.V. Pantazi [18]. However, this implies a loss of detail. It may lead the physician to click on the icon and to read the associated textual excerpt, although the excerpt may not necessarily be relevant to the patient. The general nature of some of the VCM icons can lead to the same problem. Consequently, the specificity of the overview provided by "Mister VCM" can be low. However, when a pictogram of "Mister VCM" is gray, the physician can be certain that the monograph contains no information about the corresponding anatomical or functional location, without having to read the text.

In the field of information visualization, several methods have been proposed to ease the reading of a text, such as using a document lens [19] and greeking [20]. These methods are independent of the knowledge expressed by the text, and they only highlight the structure or the aspect of the text, but not its content. These approaches, thus, are unlike "Mister VCM" which relies on the overview+detail technique, which consists of showing an overview of the whole monograph, and then displaying details on demand. Another technique is the deforming Fisheye, which deforms the document in order to show more detail from some areas. However, it has been demonstrated that the overview + detail method obtains better results when applied to texts [21]. The overview + detail model used in "Mister VCM" is particularly efficient because the overview has fixed dimension and systematically presents the same kind of information at the same location, which allows the physician to find the right information quickly by memorizing these locations.

DOPAMINE [22] is another tool, developed by Wroe *et al.*, for visualizing drug knowledge. It uses tables to represent drugs (columns) and their properties (lines), with various levels of granularity for both drugs and properties; thus, for example drugs can be collapsed into therapeutic classes. However, DOPAMINE was designed for verifying and authoring the Galen Drug Ontology, but not for clinical application. DOPAMINE relies on information visualisation techniques like table lens, which are not drug-specific; on the contrary, "Mister VCM" is based on anatomical and etiological considerations specific to the medical domain and therefore, in our opinion, this approach is more interesting for interface destined to physicians. However, the DOPAMINE table lens approach could be interesting for building a multiple-drug interface with "Mister VCM", in order to help the physician to compare the properties of several similar drugs, *e.g. *the various antihypertensives.

### About the evaluation

We demonstrate that physicians obtained the information they need from the drug monographs more rapidly and accurately with "Mister VCM" than with a textual interface. Using "Mister VCM", the response time depended only weakly on the monograph length; this was presumably due to the fixed dimensions of "Mister VCM". A longer text implies more icons on "Mister VCM", but the size of "Mister VCM" itself is unchanged. In addition, the response time with "Mister VCM" is independent of the question type. "Mister VCM" represents implicit knowledge explicitly, so it provides explicit replies to all questions. By contrast, when using texts, questions with implicit replies require significantly more time. The severity of the errors recorded with the textual or the graphical interface was not significantly different.

We applied an evaluation methodology designed to avoid some of the bias which could have compromised the evaluation process. As the monographs were fictitious, the physicians could not use their personal knowledge to answer the questions. In addition, the monographs were generated at random, and therefore the designers of "Mister VCM" did not influence the monograph choice. Finally, the randomized order of questions prevents from a potential order effect on the results.

Several criteria can be used for evaluating the efficiency of drug information database interfaces, *e.g. *the number of clicks required to find the response. However, response time seems to be the most widely used and recommended criterion in the field of human-computer interactions [11].

For the evaluation, we compared the "Mister VCM" graphical interface to a textual interface which organizes the text into four sections: contraindications, drug interactions, cautions for use, adverse effects. This textual presentation is justified by the fact that it is the most basic and common way used in drug dictionaries for presenting drug monographs to physicians. However, as "Mister VCM" acts like an index, it would also have been possible to use a more sophisticated textual interface incorporating an index. However, it is difficult to determine precisely what is the reference indexed textual interface.

### Using "Mister VCM" in real life

The automatic generation of "Misters VCM" for drug monographs was possible thanks to the Theriaque indexed data and the manual mapping of the Theriaque thesaurus to VCM [8]. Similar automatic generation of "Misters VCM" could be performed for any drug monograph-like document provided that the document is segmented into a set of sentences, each of them being indexed by the relevant terms (which is the case in Theriaque). The classifications or thesauri used for indexing the document must be manually mapped in VCM. This mapping is much easier if the classification provide "is-a-kind-of" relations (such as CIM10 or ATC): in this case, only the more general terms of the classification need to be mapped, and then inheritance can be used to associate an icon to the more precise terms, since VCM is not precise enough to distinguish all terms.

The current version of "Mister VCM" shows the information from the monograph of a single drug. However, the interface may be extended to visualize several drugs, either for comparing drugs, or for regrouping the drugs of a given prescription.

## Conclusion

The results obtained with "Mister VCM" seem very promising for facilitating physicians' access to drug monograph content relevant to the specificities of individual patients. This interface now requires evaluation in physicians' routine practice. This could lead to identify limits of "Mister VCM" and the VCM language which could be enriched in future versions.

Application to formats other than computer-based systems may also be possible. In particular, the fixed dimension of "Mister VCM" makes it suitable for Personal Digital Assistants (PDA). "Mister VCM" could also be useful for printed documents, like desktop references, because it shows explicitly the absence of information related to a given anatomical or functional system.

Other applications are foreseen. This type of interface, where a graphical overview of the content is presented to the user, could be applied to other types of medical document including clinical guidelines. This approach could be also used in the domain of electronic patient records. We are currently developing similar applications to synthetize the patient diseases, risks, and treatments.

## Competing interests

VCM and "Mister VCM" are protected by a patent, taken by University Paris 13. They are intended to be freely available for academic uses.

## Authors' contributions

J-BL, CD and AV designed the graphical interface. J-BL implemented the prototype. J-BL, CD, AV and PO designed the evaluation. AB–H performed the statistical analysis. J-BL, CD, AV and AB–H wrote the manuscript. All authors read and approved the final manuscript.

## Pre-publication history

The pre-publication history for this paper can be accessed here:


